# Production and purification of outer membrane vesicles encapsulating green fluorescent protein from *Escherichia coli*: a step towards scalable OMV technologies

**DOI:** 10.3389/fbioe.2024.1436352

**Published:** 2024-11-14

**Authors:** Julian Daniel Torres-Vanegas, Nicolas Rincon-Tellez, Paula Guzmán-Sastoque, Juan D. Valderrama-Rincon, Juan C. Cruz, Luis H. Reyes

**Affiliations:** ^1^ Grupo de Diseño de Productos y Procesos (GDPP), Department of Chemical and Food Engineering, Universidad de Los Andes, Bogota, Colombia; ^2^ Department of Biological Sciences, Universidad de Los Andes, Bogota, Colombia; ^3^ Department of Biomedical Engineering, Universidad de Los Andes, Bogota, Colombia; ^4^ Grupo GRESIA, Department of Environmental Engineering, Universidad Antonio Nariño, Bogota, Colombia

**Keywords:** outer membrane vesicles, green fluorescent protein, protein encapsulation, cell immobilization, size exclusion chromatography (SEC)

## Abstract

Outer membrane vesicles (OMVs) are spherical structures that contain a small fraction of the periplasm of Gram-negative bacteria, surrounded by its outer membrane. They are naturally produced and detached from the bacterial surface, participate in diverse biological processes, and their diameter size is in the range of 10–300 nm. OMVs have gained interest in different applications, such as the development of biosensors, vaccines, protein chips, and the encapsulation of heterologous proteins and peptides expressed by these microorganisms. However, the use of OMVs in these applications is limited due to the low yields and high purification costs. In this study, we produced green fluorescent protein (GFP) encapsulated into OMVs using *Escherichia coli* JC8031 transformed with pTRC99A-ssTorA-GFP to establish the production and purification route. Results showed that the motility of the strain prevents its immobilization in alginate, which hampers the purification of OMVs. To address this issue, a zeolite-based column was used to chromatographically separate the OMVs from smaller particles. Further experiments will be focused on standardizing the production and purification of OMVs at a scalable level.

## 1 Introduction

The intersection of biotechnology and micro-nanobiotechnology represents a rapidly expanding frontier in science and technology, driving innovation across a wide range of industries (Shahcheraghi et al., 2022; Rodríguez et al., 2024b; Soni et al., 2023; Rodríguez, Andrade-Pérez, et al., 2023). In biomedical engineering, micro-nanobiotechnology is fueling the development of advanced cellular models (Guzmán-Sastoque et al., 2024), diagnostic tools, and targeted therapies (Ortseifen et al., 2020; Rodriguez et al., 2021; Elkodous et al., 2023). Similarly, in chemical engineering, it enables the creation and purification of novel materials with enhanced properties for various processes (Zhao et al., 2020; Li et al., 2020; Rodríguez, Guzmán-Sastoque, et al., 2023). The food science industry is also reaping benefits from these advancements, particularly in food safety, preservation, and nutritional enhancement.

At the core of these innovations is the synthesis and purification of novel nanomaterials (Rodríguez et al., 2024a). These materials are crucial for numerous applications, ranging from medical devices and drug delivery systems to industrial catalysts and environmental sensors. The ability to precisely manipulate these materials at the nanoscale is opening new possibilities and pushing the boundaries of what can be achieved in these diverse fields. A notable example of such advancements is the study and application of outer membrane vesicles (OMVs), which are naturally produced by Gram-negative bacteria.

Outer membrane vesicles (OMVs) are structures produced by Gram-negative bacteria that consist of a portion of the periplasm surrounded by a small part of the outer membrane (Schwechheimer and Kuehn, 2015). These vesicles range from approximately 10–300 nm in diameter and are mainly composed of phospholipids, outer membrane proteins, and lipopolysaccharides, but they may contain any molecule present in the periplasm (e.g., proteins, nucleic acids, metabolites, etc.) (Roier et al., 2016). OMVs participate in several biological processes, mainly as a secretion and delivery system for the dissemination of bacterial products, and interaction with the environment and other bacteria (Kulp and Kuehn, 2010). The multiple roles of OMVs can be attributed to the protection given by the lipidic membrane against external factors like nucleases, proteases, and pH and temperature fluctuations (Dorward and Garon, 1990; Schwechheimer and Kuehn, 2015; Hoy et al., 2010). Therefore, OMVs can be engineered beyond their natural functions for macromolecule production and particularly recombinant proteins expressed in the periplasm.

Traditionally, the main biotechnological application of OMVs has been their use as adjuvants for the development of vaccines due to their natural immunogenic characteristics (Qing et al., 2019), which can be engineered to express certain antigens and reduce toxicity (Gerritzen et al., 2017). Besides their potential as adjuvants, OMVs have also been studied as tools for tumor treatment, drug delivery, and biological imaging (Qing et al., 2019). Beyond medical uses, OMVs potential can be expanded to the generation of biosensors, protein chips, or bioreactors (Colletier et al., 2002), owing to the fact that they can provide a protection environment for fragile enzymes, avoiding conformational changes that may lead to activity loss.

The production of OMVs with high yields under laboratory conditions requires the use of bioengineered bacterial strains since most studied Gram-negative bacteria are not naturally hypervesiculating organisms (Valderrama and Gutierrez, 2018). Regarding this, several studies have reported successful approaches to generate hypervesiculating bacteria: *gna33* knockout in *Neisseria meningitis* (Ferrari et al., 2006), deletion/repression of the VacJ/Yrb ABC transport system in *Haemophilus influenzae* and *Vibrio cholerae* (Roier et al., 2016), deletion of *tolR* and *galU* in *Shigella sonnei* (Scorza et al., 2012), *nlpI* and *degS* disruptions in *E. coli* (McBroom et al., 2006), and modifications in the tol-pal pathway in *Escherichia coli*, *Shigella flexneri* and *Salmonella enterica* (Scorza et al., 2008; Bernadac, Gavioli, Lazzaroni, Raina, and Lloubès, 1998). In line with this, the deletion of tolR in *E. coli* has been shown to generate overproduction of OMVs without loss of membrane integrity (Scorza et al., 2012). Despite these advances, one of the biggest challenges for all OMVs applications are low yields and the presence of undesired macromolecules such as LPS (Lipopolysaccharides) and porins (Balhuizen, Veldhuizen, and Haagsman, 2021).

The gold standard for OMV purification consists of sequential steps of filtration and centrifugation followed by ultracentrifugation to concentrate the vesicles (Corso et al., 2017; Valderrama and Gutierrez, 2018). Since ultracentrifugation demands a considerable amount of time and resources, more cost-effective purification methods are necessary to scale-up OMV-based processes. In addition, ultracentrifugation has the downside of damaging the vesicles and leading to aggregation, which affects subsequent applications (Corso et al., 2017). In this report, we evaluated the encapsulation of GFP in OMVs produced by *E. coli*. Results showed low encapsulation efficiencies due to the motility of the bacterium. This leads to failures in the immobilization of the microorganism in alginate and prevents the purification of OMVs. Therefore, a new method for the purification of OMVs was implemented under laboratory conditions. This method implies the use of a zeolite-based column that enables us to separate OMVs from smaller particles.

## 2 Materials and methods

### 2.1 Bacterial strain and plasmid

The strain *E. coli* JC8031 transformed with the plasmid pTRC99A-ssTorA-GFP (Supplementary Figure S1A) was obtained from the Chemical Engineering Department at Universidad de los Andes. The plasmid sequence can be found in the Supplementary Material. The strain exhibited deletions in TolA and TolR, thereby endowing it with the characteristic of being a hypervesiculating bacterium (Bernadac et al., 1998). Stored JC8031 was reactivated by thawing the −80°C stock on ice for 5 minutes. Then, 100 µL of stock were added to 5 mL of LB broth and LB agar plate, both supplemented with 100 μg/mL of ampicillin (Merck KGaA, Darmstadt, Germany), which were cultured at 37°C for 12 h. The reactivated culture was maintained in LB agar at 4°C with 100 μg/mL of ampicillin.

The motility assay was conducted on soft agar plates containing 0.5% (w/v) agar and 100 μg/mL of ampicillin. For this assay, a pre-inoculum of *E. coli* JC8031 was grown overnight in LB broth with 100 μg/mL of ampicillin at 37°C and 250 rpm. The culture was then diluted to reach an OD600 of 0.4, and 6 μL of the culture was spotted onto the soft agar plate, followed by incubation at 37°C for 18 h. *Staphylococcus aureus* ATCC25923 was also used in motility assays as a control. After incubation, motile bacteria were isolated and inoculated onto soft agar plates to observe motility. All experiments were performed in triplicate.

### 2.2 Plasmid purification and restriction enzyme digestion

To verify that pTRC99A-ssTorA-GFP was effectively present in the bacteria, plasmidic DNA was extracted using the Monarch^®^ Plasmid Miniprep Kit (New England Biolabs). Next, the extracted plasmid was digested with EcoRI and HindIII following the manufacturer’s instructions (New England Biolabs). Finally, the obtained plasmid was separated and visualized via agarose gel electrophoresis (Supplementary Figure S1B).

### 2.3 Confocal microscopy analyses

A JC8031 pre-inoculum was grown overnight in LB broth supplemented with 100 μg/mL of ampicillin at 37°C and 250 rpm. The following day, 1 mL of the pre-inoculum was transferred into 10 mL of fresh LB Lennox broth containing 100 μg/mL of ampicillin in a sterile 15 mL conical tube. The culture was incubated at 37°C and 250 rpm until it reached an OD_600_ of 0.4. To induce GFP expression, IPTG was added to a final concentration of 1 mM. After 4 h, the induced cells were fixed on glass coverslips coated with 0.4% agarose and visualized using an Olympus FV1000 confocal laser scanning microscope with a 490 nm excitation filter and a 520 nm emission filter.

For membrane localization, JC8031 cells were grown under the same conditions as described above. GFP expression was induced with 1 mM IPTG, and after 4, 28, and 72 h of induction, the cells were fixed on glass coverslips coated with 0.4% agarose. Membranes were visualized using DiI (1,1′-Dioctadecyl-3,3,3′,3′-Tetramethylindocarbocyanine Perchlorate) dye, and imaging was performed on the Olympus FV1000 confocal laser scanning microscope with a 550 nm excitation filter and 564 nm emission filter.

### 2.4 Protein expression induction

A JC8031 pre-inoculum was grown overnight at 37°C and 250 rpm in LB broth supplemented with 100 μg/mL of ampicillin. Then, 1 mL of pre-inoculum was added to 50 mL of fresh LB broth supplemented with 100 μg/mL of ampicillin, previously pre-warmed at 37°C, using a 150 mL Erlenmeyer flask. This culture was grown at 37°C (degrees Celsius) and 250 rpm until reaching an OD_600_ of 0.4. Next, IPTG was added to a final concentration of 1 mM to induce GFP expression. The induced culture was grown at 37°C and 10 × g to produce OMVs loaded with GFP.

After 24 h, the induced JC8031 culture was centrifuged at 2,100 *g* for 10 min. The bacterial pellet was discarded, and the supernatant was filtered through a 0.45 µm syringe filter (PVDF filter membrane, 13 mm diameter) to eliminate any remaining bacteria. Then, the filtered supernatant containing the OMVs was concentrated three times using a 300,000 MWCO centrifuge filter. Each time, the collected LB broth was discarded, and 1X PBS (NaCl: 137 mM, KCl: 2.7 mM, Na_2_HPO_4_: 10 mM, KH_2_PO_4_: 1.8 mM, 7.4 pH) was added to the retentate. Additionally, 100 µL of concentrated OMVs were cultured for 24 h in LB agar 1.5% supplemented with 100 μg/mL of ampicillin to verify the complete elimination of living bacteria.

### 2.5 OMVs characterization

#### 2.5.1 Size Distribution and Morphology

The size distribution of outer membrane vesicles (OMVs) was measured using a Zetasizer Nano ZS (Malvern Panalytical, Malvern, UK). Two 10% v/v dilutions of concentrated OMVs in water were sonicated for 30 s using a Branson 2,800 Series ultrasonic cleaner (Danbury, CT, United States) to prevent agglomeration. One of these dilutions was additionally treated with 1% v/v Triton X-100 (Sigma-Aldrich, St. Louis, MO, United States) to disrupt the vesicles and analyze their response to detergent treatment.

The morphology and size of the OMV were further examined using transmission electron microscopy (TEM) with a Tecnai F20 instrument operating at 30 kV (FEI Company, Fremont, CA, United States). For TEM analysis, OMVs were washed twice with 0.1X PBS by centrifugation at 17,000 × g for 1 h per wash, then concentrated in 20 µL of 0.9X PBS. Samples were fixed for 20 min with glutaraldehyde (3:1 ratio with 0.9X PBS), followed by two washes with 0.9X PBS and a final concentration in 20 µL of 0.9X PBS.

#### 2.5.2 Fluorescence Analysis

To evaluate the integrity and protein content of OMVs, two 10% v/v dilutions of OMVs from induced and non-induced cultures were treated with proteinase K (ThermoFisher Scientific, Waltham, MA, United States) or 1% v/v Triton X-100. Proteinase K treatment was used to digest proteins, while Triton X-100 was used to disrupt the vesicles. Fluorescence measurements were conducted using a FluoroMax^®^ spectrofluorometer (HORIBA Scientific, Kyoto, Japan).

To confirm proteinase K activity, induced JC8031 cells were grown, centrifuged at 11,500 × g for 30 min, and the bacterial pellet was resuspended in 1X PBS. The cells were then treated with 1% v/v Triton X-100 and sonicated for 20 min at room temperature to lyse the cells. The solution was centrifuged at 11,500 × g for 30 min to recover the supernatant containing concentrated GFP. Spectrofluorometric analysis was performed on GFP solutions with and without 1.68 μg/μL proteinase K to assess its effect on GFP fluorescence.

### 2.6 OMVs purification via size exclusion chromatography (SEC)

A JC8031 pre-inoculum was grown overnight at 37°C and 250 rpm in LB broth supplemented with 100 μg/mL of ampicillin. Then, 1 mL of pre-inoculum was added to 50 mL of M9 medium (200 mL of 5X M9 salts solution (NaCl, Na_2_HPO_4_, KH_2_PO_4_, NH_4_Cl), 800 mL of ddH_2_O, 2 mL of 1M MgSO_4_ solution, 0.1 mL of 1M CaCl_2_ solution, and 10 mL of 40% w/v glucose) supplemented with 100 μg/mL of ampicillin. This medium was chosen for its specificity in promoting the growth of *E. coli* under conditions of slow growth rate while still allowing for protein expression induction. This culture was grown at 37°C and 250 rpm until it reached an OD_600_ of 0.4. IPTG was then added to a final concentration of 1 mM to induce GFP expression. The induced culture was grown for 72 h to produce OMVs loaded with GFP.

Supplementary Figure S2 shows an overview of the procedure for the production and purification of OMVs. SEC was conducted with a Poly-Prep gravity column (Bio-Rad Laboratories, Hercules, California, United States) packed with 1 g of a sterilized solid mixture with a 70/30 ratio of zeolite (700 mg of zeolite and 300 mg of glass beads) (ZEODET325EC, Grupocelta, Bogotá, Colombia) and 0.5 mm glass beads (United States Scientific, Ocala, Florida, United States), respectively. Meanwhile, the induced culture was centrifuged at 2,100 *g* for 10 min. The bacterial pellet was discarded, and the supernatant was filtered with a 0.45 µm syringe filter to eliminate the remaining bacteria. Subsequently, 2 mL of the supernatant was loaded onto the column equilibrated with 1X PBS, and four continuous flow fractions of 0.5 mL each were collected via centrifugation at 100 *g*, with the column placed within a 50 mL conical tube. The collection of the liquid continued until reaching a volume of 0.5 mL per fraction. Four additional 0.5 mL fractions were collected after adding 2 mL of 1X PBS to the column. A major drawback of this procedure is that the employed zeolite has a wide range of particle sizes, some of which might be easily released from the column during operation. Consequently, prior to downstream analysis of the fractions, it was necessary to centrifuge the fractions to eliminate zeolite particles that could interfere with fluorescence and size distribution measurements. All the fractions were centrifuged for 2 min at 16,000 × g to precipitate swept zeolite particles, and then they were processed as described previously to measure size and fluorescence. This step in our proposed method does not occur over a considerable period of time but could potentially lead to the rupture and aggregation of OMVs. However, in general, the rupture and damage of OMVs occur at speeds greater than 100,000 xg. Protein concentration in each fraction was measured using a NanoDrop^®^ ND-1000 UV-Vis Spectrophotometer (ThermoFisher Scientific, Waltham, Massachusetts, United States) at 280 nm, using 1X PBS as a blank. Moreover, the recovery percentage of GFP-loaded OMVs was calculated as the fluorescence intensity (cps) in a fraction divided by the fluorescence intensity of the sample before processing.

In addition, proteins from all fractions and from the JC8031 pellet were visualized via SDS-PAGE following the BioRad protocol (Bio-Rad, 2012) to verify the previous results, using a 4% storage gel, a 10% separation gel, and a 1X Tris-glycine running buffer. Total protein staining was done either with Coomassie Brilliant blue or with silver staining if the protein concentration was not high enough for detection.

For silver staining, the protocol provided by the Proteomics Resource Center at The Rockefeller University was followed. Four main steps were performed. Initially, fixation was conducted for 20 min using a solution comprising 50% *v/v* methanol (Sigma-Aldrich, St. Louis, MO, United States) and 5% *v/v* acetic acid (Sigma-Aldrich, St. Louis, MO, United States). Subsequently, the sample underwent a 10-min wash with a 50% *v/v* methanol solution, followed by a 10-min rinse with Type 2 water. Sensitization was then achieved by exposure to 0.02% *w/v* pentahydrated sodium thiosulfate (Sigma-Aldrich, St. Louis, MO, United States) for 1 min, followed by two 1-min washes with Type 2 water.

Following this, a solution containing 0.1% *w/v* silver nitrate (Sigma-Aldrich, St. Louis, MO, United States) and 0.08% *v/v* formaldehyde (36%) (Sigma-Aldrich, St. Louis, MO, United States) was added for 20 min. A 1-min wash with Type 2 water ensued, after which a solution of 2% *w/v* sodium carbonate (Merck KGaA, Darmstadt, Germany) with 0.04% *v/v* formaldehyde (36%) was introduced for revelation. Finally, the reaction was halted with a 5% *v/v* acetic acid solution for 10 min, followed by a 5-min rinse with Type 2 water.

In the specific case of vesicle disruption before and after purification, 1% v/v Triton X-100 was utilized. Only fraction #4 of the SEC purification was visualized, as it contained the highest protein concentration.

### 2.7 Statistical analysis

The statistical significance of differences among experimental groups was assessed using a One-Way ANOVA, followed by Tukey’s *post hoc* test for Figure 3C, and a t-test for Figure 3D, as performed with GraphPad Prism Software version 10.2 (GraphPad Software Inc., Boston, MA, United States). A *p*-value of less than 0.05 was considered statistically significant.

## 3 Results and discussion

### 3.1 Confocal microscopy

**FIGURE 1:**
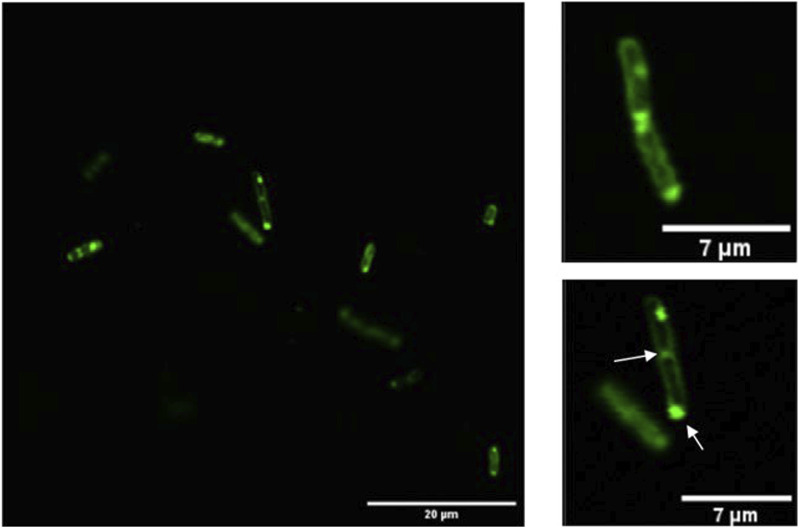
Confocal microscopy of induced *Escherichia coli JC8031*. The figure shows GFP fluorescence primarily localized at the cell periphery, indicating successful secretion into the periplasm. Arrows mark the inclusion bodies, which are concentrated near the cell poles, highlighting their accumulation in these regions. The inclusion bodies are noted to form in areas of macromolecular crowding, which can influence recombinant protein production.

Confocal microscopy was performed to verify the activity and export of GFP to the periplasm, with some instances showing GFP retained in the cytoplasm. Figure 1 illustrates that fluorescence is concentrated at the periphery of certain cells, indicating that GFP is correctly folded and secreted into the periplasm. These observations are consistent with previous studies demonstrating active GFP export to the periplasm of *E. coli* via the TAT pathway (Thomas et al., 2001; Santini et al., 2001), which reported 46% and 65% of total-cell GFP fluorescence localized to the periplasm.

Inclusion bodies, primarily located near cell poles, are also observed in Figure 1. Inclusion bodies are cytoplasmic protein aggregates that naturally occur in prokaryotic cells but are more prevalent when recombinant proteins are expressed in bacteria (Lee et al., 2008). They consist of insoluble protein molecules that progressively form aggregates (Rinas et al., 2017). The formation of these bodies can hinder recombinant protein production as they contain misfolded and inactive polypeptides (Wu et al., 2011). Such structures are commonly found at the poles of *E. coli* or at future binary fission sites (Lindner et al., 2008; Winkler et al., 2010; Coquel et al., 2013). Pole localization is largely attributed to macromolecular crowding in the nucleoid region, which inhibits the formation of large protein aggregates in the central cell region (Rinas et al., 2017).

**FIGURE 2:**
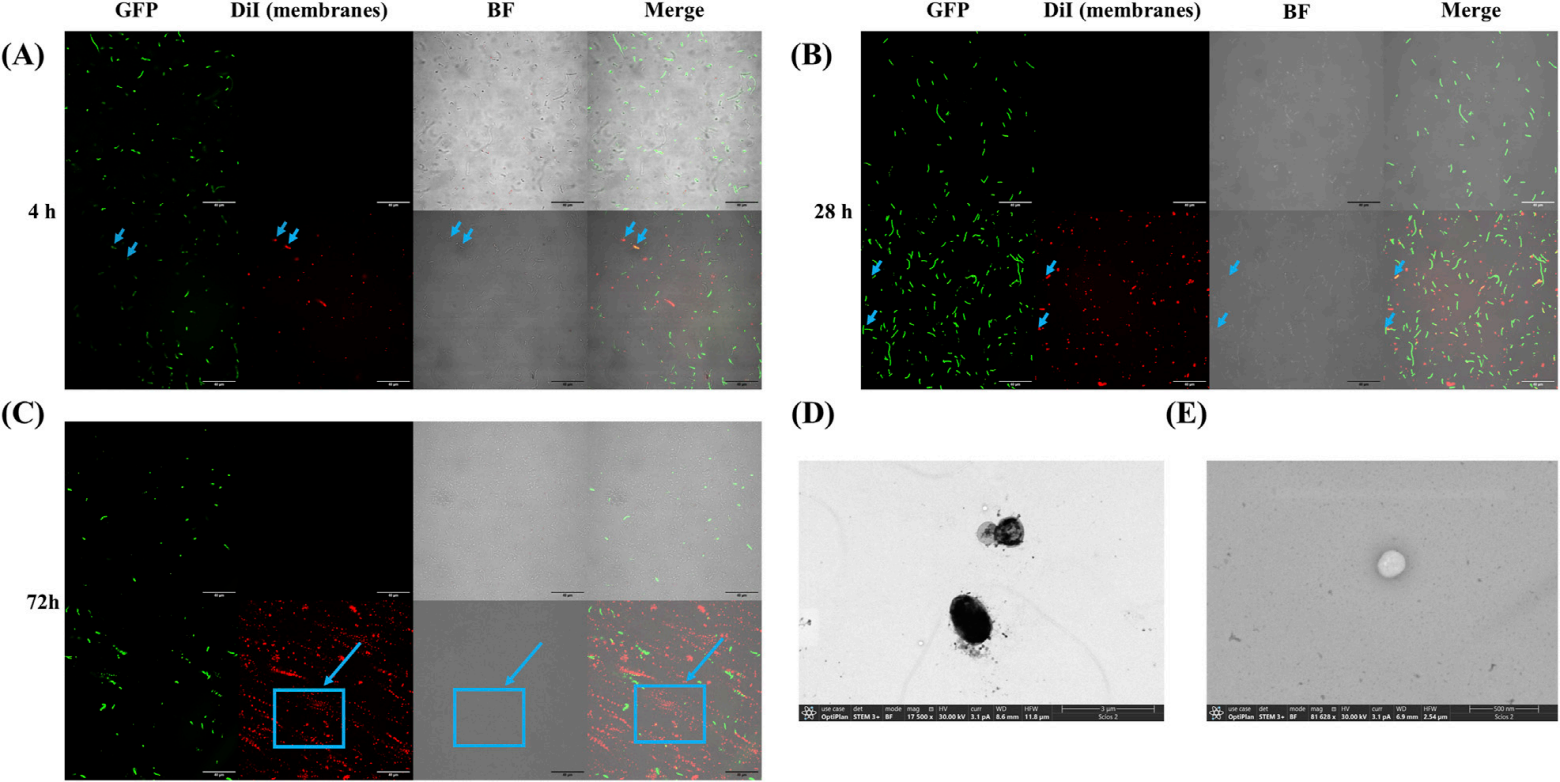
Localization of GFP and membrane staining in *Escherichia coli* JC8031 and OMVs. **(A)** GFP and membrane localization after 4 h of IPTG-induced GFP expression. Arrows indicate that only bacteria undergoing vesiculation at the time of imaging exhibit overlapping fluorophores. **(B)** GFP and membrane localization after 28 h of IPTG-induced GFP expression. Similar to panel **(A)**, only vesiculating bacteria show intersecting fluorophores. **(C)** GFP and membrane localization after 72 h of IPTG-induced GFP expression. The arrow highlights a region with increased DiI fluorophore intensity, suggesting a higher density of membranes. **(D)** TEM image of the sample before purification, showing numerous artifacts likely due to the lack of purification. **(E)** TEM image of OMV after purification.

The visualization of GFP expression diminishes over time due to the discontinuous induction of expression, leading to a stationary phase. Additionally, some bacteria exhibit periplasmic GFP expression, while others show cytoplasmic localization (Figures 1, 2A–C). This variation may be related to the absence of specific signal sequences for proper GFP localization. Moreover, bacteria displaying membrane-associated signals were identified at the time of imaging, as indicated by the arrows. Finally, an increase in DiI fluorescence intensity, a membrane marker, was observed over time, suggesting a higher presence of membrane structures without necessarily implying an increase in vesiculation.

### 3.2 OMVs characterization

**FIGURE 3:**
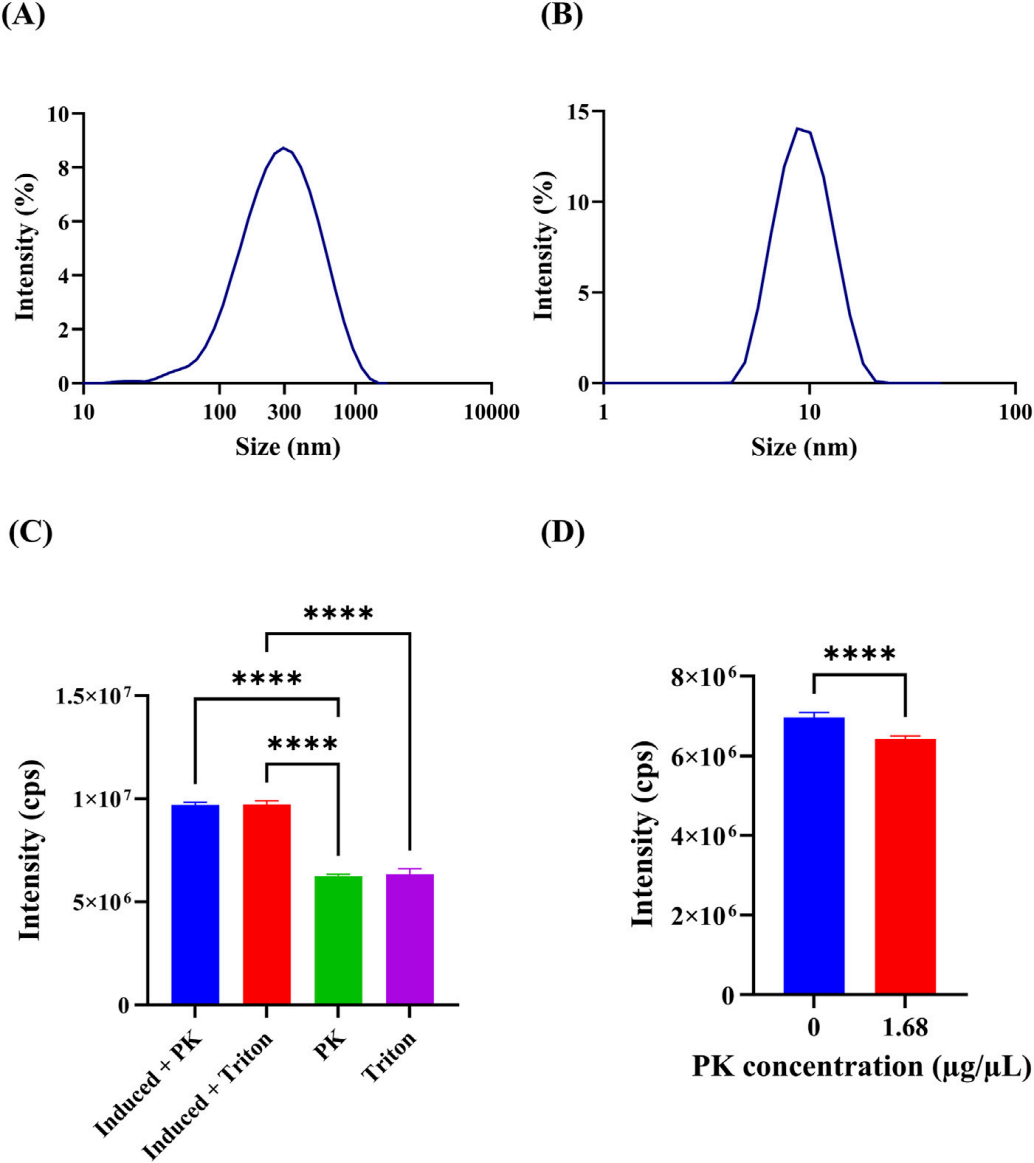
Characterization of OMVs. **(A)** Size distribution of OMVs from induced bacteria, as measured by Dynamic Light Scattering (DLS). This provides insight into the typical size of OMVs produced under induction conditions. **(B)** Size distribution of OMVs from induced bacteria treated with 1% v/v Triton X-100. The treatment likely disrupts the OMVs, causing a reduction in size and indicating changes in OMV structure. **(C)** Spectrofluorometric analysis of OMVs subjected to different treatments to assess their fluorescence intensity. This helps evaluate the stability and integrity of GFP encapsulated within the OMVs. Induced + PK: 1 × 10⁷ cps ±127,597 cps, Induced + Triton X-100: 1 × 10⁷ cps ±166,118 cps, PK: 6,247,143 cps ±92,864 cps, Triton X-100: 6,328,571 cps ±282,927 cps. ANOVA and Tukey’s test were conducted to determine statistical significance. ** indicates *p*-values <0.01, *** *p*-values <0.001, and **** *p*-values <0.0001. **(D)** Spectrofluorometric analysis of GFP solutions with and without proteinase K (PK) treatment. This comparison assesses the effectiveness of PK in affecting GFP fluorescence, indicating the protective capability of OMVs against proteolytic degradation. 0 μg/μL: 6,962,857 ± 127,765, 1.68 μg/μL: 6,422,857 ± 76,095. Arrows in the figure highlight key features, such as the size range of OMVs and the effects of different treatments on fluorescence intensity. t-test was conducted to determine statistical significance. ** indicates *p*-values <0.01, *** *p*-values <0.001, and **** *p*-values <0.0001. We used three biological replicates, and each measurement was performed in triplicate.

DLS measurements enabled us to determine that purified OMVs had an average hydrodynamic diameter of 314.5 nm (polydispersity index (PI) of 0.21%) (Figure 3A), which is consistent with previous reports (Roier et al., 2016; Kim et al., 2017). Roier et al. and Kim et al. also measured the diameter of OMVs using a ZetaSizer. However, it is important to note that these authors utilized bacterial strains with different mutations compared to the strain employed in this study. Additionally, after treating OMVs with the detergent Triton X-100 (Figure 3B), the average size distribution by intensity reduced to 12.32 nm (PI of 0.77%); which likely indicates disruption of OMVs structure and agglomeration of free outer membrane components into smaller clusters. Furthermore, no colonies appeared after plating concentrated OMVs, confirming that no viable *E. coli* cells were observable after purification.

Furthermore, TEM analysis revealed that the OMVs exhibited a uniform spherical morphology, with no residual bacterial contaminants observed after purification (Figure 2E). The micrographs indicated an average OMV diameter of 200 nm, consistent with previous studies (N. Li et al., 2024) (Li et al., 2023).

The presence of particles with a size larger than 400 nm, as observed in Figure 3A, which is far from the OMVs size range reported in other investigations, may be the result of contamination with bacterial debris. Previous studies have demonstrated that tolRA gene deletion in *E. coli* results in substantial overproduction of OMVs without loss of membrane integrity (Bernadac, Gavioli, Lazzaroni, Raina, and Lloubès, 1998; Scorza et al., 2012; Scorza et al., 2008).The Tol-Pal system is a multiprotein complex found in Gram-negative bacteria that is involved in processes such as the rearrangement of the peptidoglycan layer at division sites, maintenance, anchoring to the peptidoglycan layer, and invagination during cell division of the outer membrane (Bouveret et al., 1995; Dennis, Lafontaine, and Sokol, 1996; Gerding et al., 2007; Szczepaniak et al., 2020). It is known that mutations in tol-pal genes lead to increased sensitivity to antibiotics and detergents and leakage of periplasmic components (Gerding et al., 2007), as well as an increase in the phospholipids-to-lipopolysaccharides ratio in the outer membrane, making it more susceptible to mechanical stress (Rottem and Leive, 1977; Jefferies, Shearer, and Khalid, 2019). This could partially explain the presence of particles larger than the expected size for OMVs that correspond to outer membrane debris released during centrifugation.

Moreover, some investigations have reported blebbing of the outer membrane from the septal region during cell division in tol-pal mutants, which occurs because the Tol-Pal complex plays an important role in proper outer membrane and cell wall processing (Weigand and Rothfield, 1976; Gerding et al., 2007; Petiti et al., 2019; Szczepaniak et al., 2020; Yakhnina and Bernhardt, 2020). Gerding et al. (2007), who used fluorescence microscopy and found that tol-pal mutants exhibited outer membrane blebs larger than 300 nm, suggested that these structures initially form at the division site and then are inherited by new poles upon complete binary fission, or might be released forming OMVs. Although Figure 1 shows no clear expansion of the periplasmic space at division sites or OMVs, the cell in Figure 1C appears to have a polar bleb that is consistent with the findings made by Gerding et al. (2007) and Petiti et al. (2019), who used fluorescence microscopy and inverted epifluorescence microscopy, respectively. Therefore, the OMVs larger than 300 nm identified here (Figure 3A) are likely to be derived from division site blebs. Since JC8031 lacks tolRA, it produces not only larger OMVs but also an enhanced capacity to accumulate periplasmic GFP due to a reduced interaction between the outer and inner membranes that reduces their proximity, particularly at constriction sites (Walburger, Lazdunski, and Corda, 2002; Yakhnina and Bernhardt, 2020). Owing to such functions, the Tol-Pal system has been proposed as a mechanism involved in the regulated production of OMVs in wild-type bacteria (Szczepaniak et al., 2020). Looking forward to improving the yield of OMV protein encapsulates, it remains to be determined if the Tol-Pal and Tat systems interact with each other.

Four different treatments were carried out to determine if GFP was successfully encapsulated inside the OMVs. As shown in Figure 3C, OMVs from induced JC8031 treated with proteinase K (PK) or Triton X-100, produced the same fluorescence intensity level. Similarly, OMVs from non-induced bacteria after the same treatments led to the same fluorescence level. However, their intensity was almost one order of magnitude smaller than that of vesicles from induced bacteria. Since the fluorescence intensity of both disrupted (i.e., treated with Triton X-100) and protease-treated OMVs is the same, it can be stated that such vesicles effectively protect their load from the external environment, as reported by Dorward and Garon (1990) and Hoy et al. (2010). Moreover, the significant basal expression of GFP may be explained by the functioning of lac operon repression, which is modulated by DNA supercoiling, as has been demonstrated by Fulcrand et al. (2016). Under high glucose concentrations, *E. coli* produces enough ATP for normal functioning; in particular, DNA gyrase is fully active; hence, DNA is likely to be in a more supercoiled state (Fulcrand et al., 2016). This condition promotes stronger repression, thus preventing basal expression. On the contrary, a lack of glucose reduces supercoiling activity, which results in weaker repressor binding and increased basal expression (Fulcrand et al., 2016). Therefore, the lack of glucose in the LB broth could explain why GFP is produced even in the absence of IPTG induction. Furthermore, the trc promoter is known for having a leaky transcription in the absence of induction (Tegel et al., 2011).

Moreover, an additional assay was executed to confirm proteinase K (PK) activity against GFP in a concentrated sample of OMVs. Although it was observed that the difference in fluorescence intensity between GFP in the presence and absence of PK is significant (Figure 3D) (t-test *p*-value <0.05), the difference in the total intensity was not considerable. This could be explained by a very high concentration of GFP (not measured) that resulted in a low PK/GFP, which limited protease activity. GFP is known for being highly resistant to proteolysis by various proteases owing to its β-barrel structure, and it can remain active even when it is cleaved (Chiang et al., 2001; Aoki et al., 2008). Consequently, another explanation for the observed results is that either PK failed to cleave GFP, or it showed no impact on its fluorescence activity.

### 3.3 OMVs purification using size exclusion chromatography

**FIGURE 4:**
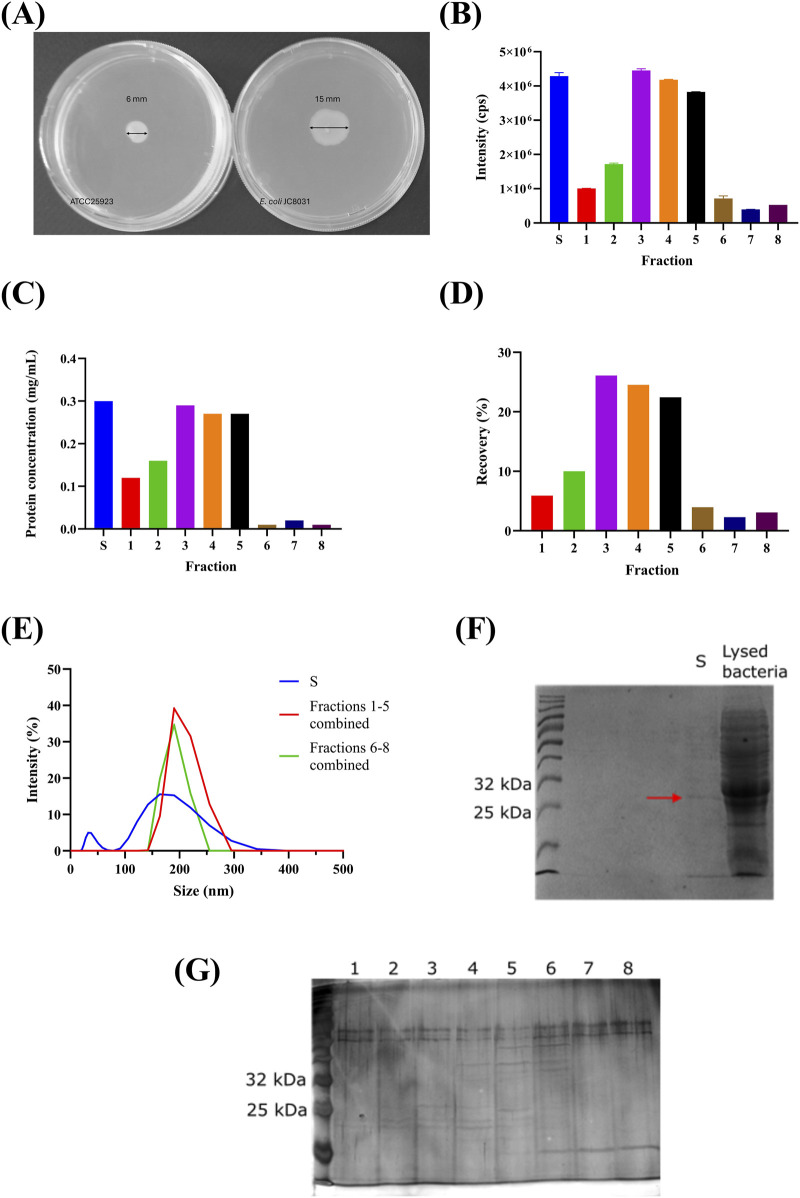
Purification Stage Analysis. **(A)** Motility Test: Comparative Analysis of *Escherichia coli* JC8031 and *Staphylococcus aureus* ATCC25923 in Soft Agar Assays, confirming that *Escherichia coli* JC8031 can move through the alginate beads. **(B)** Spectrofluorometric analysis of SEC fractions showing fluorescence intensity. S: 4,288,343 cps ±102,018 cps, 1: 1,005,943 cps ±5.260 cps, 2: 1,719,463 cps ±26,776 cps, 3: 4,450,423 cps ±50,005 cps, 4: 4,181,993 cps ±10,579 cps, 5: 3,825,490 cps ±5,072 cps, 6: 715,430 cps ±73,307 cps, 7: 393,813 cps ±5,406 cps, 8: 528,007 cps ±1,000 cps. **(C)** Protein concentration in SEC fractions is determined by A280 absorbance. **(D)** Fluorescence recovery percentage of GFP-loaded OMVs in SEC fractions, with the initial sample (S) providing a baseline for comparison (cps: counts per second). **(E)** DLS measurements of SEC fractions reveal particle size distribution. **(F)** SDS-PAGE analysis with Coomassie staining, highlighting GFP bands (indicated with a red arrow). Lane 1: Weight marker, Lane 2: None, Lane 3: None, Lane 4: S (initial sample), Lane 5: Lysed bacteria. **(G)** SDS-PAGE analysis with silver staining, showing GFP bands (indicated with a red arrow). Arrows indicate the GFP bands in SDS-PAGE gels. The figure illustrates the effectiveness of SEC in purifying OMVs and the challenges faced in achieving high encapsulation efficiency.

In an initial attempt, we encapsulated the strain in core-shell calcium-alginate beads with an approximate diameter of 3 mm and pore walls averaging less than 2 µm. The capsules were prepared using 4% w/v sodium alginate and 100 mM calcium chloride. The bacterial culture was mixed with sodium alginate in a 1:1 ratio and added dropwise to the calcium chloride solution. However, bacterial growth was observed inside the liquid medium within less than 24 h (data not shown). Despite maintaining sterile conditions during bead preparation, the bacterial growth suggests that JC8031 can actively move through the alginate matrix, as confirmed by motility test (Figure 4A). This method is a rapid, simple, and macroscopic approach to assessing bacterial motility, like techniques employed in other studies (Partridge and Harshey, 2020; Khider, Willassen, and Hansen, 2018; Tamar, Koler, and Vaknin, 2016). However, there are other microscopic techniques available, such as Differential Interference Contrast (DIC) microscopy, confocal microscopy, and Differential Dynamic Microscopy (Palma et al., 2022). Figure 4A confirms that the bacterial strain exhibits motility, as outward movement is observed at the peripheral ring of the expanding swim colony. For the motile *E. coli* JC8031 strain, a zone of opacity in the semi-liquid agar of 15 mm ± 3 mm was observed, indicating active motility due to the presence of flagella, which rendered the encapsulation of JC8031 in alginate beads ineffective. In contrast, *S. aureus* ATCC25923 showed an opacity zone of 6 mm ± 1 mm, attributed to cellular division rather than motility. A similar phenomenon was reported by Páez-Vélez, Rivas, and Dussán (2019), where *Lysinibacillus sphaericus* was continuously released from calcium alginate beads due to flagellar propulsion. This finding underscores the need for more effective purification strategies to improve OMV yield.

*Encapsulation Efficiency and Purification:* Based on TEM images, our proposed purification method effectively removes bacterial cell debris and likely eliminates lipopolysaccharides or nucleic acids, allowing for clearer and more distinct visualization of OMV compared to the unpurified sample (Figure 2D). In contrast, the unpurified sample contained various contaminants, including culture medium, bacterial cells, and lipopolysaccharides, making it difficult to differentiate OMVs from these impurities. The purified OMVs exhibit the expected morphology and remain intact, with no observed artifacts (Figure 2E).

To benchmark our method, we compared it with ultracentrifugation, a common technique for OMV purification. Ultracentrifugation is effective due to differences in size and density but has limitations, including the retention of cellular debris, potential morphological changes to OMVs, and the risk of aggregation or rupture (Reimer et al., 2021) (Li and Liu, 2020).

Our study also explored an alternative purification approach combining size-exclusion chromatography (SEC) with zeolite columns. SEC is known for its ability to yield high quantities of mammalian extracellular vesicles without altering their morphology (Böing et al., 2014; Nordin et al., 2015; Corso et al., 2017). Figure 4B shows that fractions 3, 4, and 5 exhibited higher fluorescence, corresponding to a recovery percentage of GFP-loaded OMVs of 73.06% (Figure 4D). The combined recovery percentage for the first five fractions was 88.98%. Furthermore, Figure 4C confirms that GFP concentration correlates directly with fluorescence. The particle size distribution was bimodal, with peaks below 50 nm and above 150 nm (Figure 4E). In contrast, SEC fractions displayed a single peak near 200 nm, indicating effective separation of larger OMVs from smaller particles. This likely occurred because the zeolite column captured smaller impurities while allowing larger OMVs to pass through (Corso et al., 2017). The similar size distribution in fractions six to eight compared to fractions one to five suggests that these later fractions either contain OMVs without GFP or fewer loaded vesicles, pointing to potential heterogeneity in OMV composition and load, as noted by Böing et al. (2014).

To enhance homogeneity in OMV size, future work should focus on optimizing growth conditions to identify factors that significantly impact OMV release and size. Additionally, employing 0.22 µm filtration membranes may improve size uniformity and yield more consistent results across samples (Sharif, Eftekhari, and Mohit, 2021; Alves et al., 2017).

SDS-PAGE analysis of fractions with a concentration of 0.71 ng/μL (Figure 4F) confirmed the presence of GFP, which has a molecular weight of approximately 27 kDa (Tsien, 1998; Shaner, Steinbach, and Tsien, 2005). Lane S in Figure 4F shows a faint band over 25 kDa, similar to the lysed bacteria. Lanes 3, 4, and five in Figure 4G show fainter ∼27 kDa bands compared to the initial sample, while the remaining lanes lack visible GFP bands. This suggests that the protein concentration was below the detection limit of Coomassie staining, indicating very low encapsulation efficiency.

While the transport of GFP to the periplasm was sufficient to produce some loaded OMVs, the low efficiency is likely due to Tat system saturation (Barrett et al., 2003), the formation of inclusion bodies (Figure 1), and the high proportion of outer membrane proteins, which might interfere with GFP encapsulation (Koebnik, Locher, and Van Gelder, 2000; Wang et al., 2021). Improving encapsulation efficiency may require modulating the expression of non-essential membrane macromolecules (Valderrama and Gutierrez, 2018). Once these issues are addressed, zeolite columns could offer a cost-effective purification method, with SEC serving as a high-efficiency clean-up step for large OMV volumes after initial concentration by tangential flow or centrifuge filtration (Corso et al., 2017).

Finally, as shown in Supplementary Figure S3, OMVs disrupted with 1% *v/v* Triton X-100 before and after purification revealed that while GFP (approximately 27 kDa) was encapsulated, residual *E. coli* protein was also present. The protein concentration was 1.42 ng/μL, indicating that, despite the low encapsulation efficiency, the purification process effectively removes impurities and residual bacterial proteins.

## 4 Conclusion

OMVs have emerged as potent biotechnology systems to produce recombinant proteins. However, due to low yields and the presence of undesirable macromolecules that are difficult to remove, downstream processing becomes a major economic obstacle. To overcome this major issue, we explored the use of calcium alginate beads to encapsulate a hypervesiculating strain capable of producing OMVs, thereby facilitating their cost-effective production. Our results indicate that such an approach is inconvenient due to the motility of *E. coli* JC8031 that allowed its escape from the polymeric matrix. Alternatively, we implemented a SEC approach where a column was packed with zeolite as the main separating medium. This allowed us to isolate vesicles of ∼200 nm from smaller, undesired molecules of approximately 50 nm. Although zeolite has the main disadvantage of releasing small particles that might contaminate the collected fractions, it has the potential of becoming a reliable, cost-effective, and rapid method to purify OMVs. Future work will be dedicated to developing a standardized procedure to gain insights into the technical requirements and operating conditions to scale-up the process. Additionally, important efforts must be invested toward improving the encapsulation efficiency to increase the overall yield.

## Data Availability

The original contributions presented in the study are included in the article/Supplementary Material, further inquiries can be directed to the corresponding authors.

## References

[B1] Alves N. J. Turner K. B. DiVito K. A. Daniele M. A. Walper S. A. (2017). Affinity purification of bacterial outer membrane vesicles (OMVs) utilizing a his-tag mutant. Res. Microbiol. 168 (2), 139–146. 10.1016/j.resmic.2016.10.001 27773766

[B2] Aoki T. Tsuchida S. Yahara T. Hamaue N. (2008). Novel assays for proteases using green fluorescent protein-tagged substrate immobilized on a membrane disk. Anal. Biochem. 378 (2), 132–137. 10.1016/J.AB.2008.04.022 18455491

[B3] Balhuizen M. D. Veldhuizen E. J. A. Haagsman H. P. (2021). Outer membrane vesicle induction and isolation for vaccine development. Front. Microbiol. 12 (February), 629090. 10.3389/FMICB.2021.629090 33613498 PMC7889600

[B4] Barrett C. M. L. Ray N. Thomas J. D. Robinson C. Bolhuis A. (2003). Quantitative export of a reporter protein, GFP, by the twin-arginine translocation pathway in Escherichia coli. Biochem. Biophysical Res. Commun. 304 (2), 279–284. 10.1016/S0006-291X(03)00583-7 12711311

[B5] Bernadac A. Gavioli M. Lazzaroni J. C. Raina S. Lloubès R. (1998). Escherichia coli tol-pal mutants form outer membrane vesicles. J. Bacteriol. 180 (18), 4872–4878. 10.1128/JB.180.18.4872-4878.1998 9733690 PMC107512

[B6] Böing A. N. van der Pol E. Grootemaat A. E. Coumans F. A. W. Sturk A. Nieuwland R. (2014). Single-step isolation of extracellular vesicles by size-exclusion chromatography. J. Extracell. Vesicles 3 (1). 10.3402/JEV.V3.23430 PMC4159761 25279113

[B70] Bio-Rad (2012). Stain-free imaging technology: better protein electrophoresis and quantitative Western Blotting. Available at: https://www.bio-rad.com/webroot/web/pdf/lsr/literature/Bulletin_6040.pdf .

[B7] Bouveret E. Derouiche R. Rigal A. Lloubès R. Lazdunski C. Bénédetti H. (1995). Peptidoglycan-associated lipoprotein-TolB interaction. J. Biol. Chem. 270 (19), 11071–11077. 10.1074/JBC.270.19.11071 7744736

[B8] Chiang C. F. Okou D. T. Griffin T. B. Reynold Verret C. Williams M. N. V. (2001). Green fluorescent protein rendered susceptible to proteolysis: positions for protease-sensitive insertions. Archives Biochem. Biophysics 394 (2), 229–235. 10.1006/ABBI.2001.2537 11594737

[B9] Colletier J. P. Chaize B. Winterhalter M. Fournier D. (2002). Protein encapsulation in liposomes: efficiency depends on interactions between protein and phospholipid bilayer. BMC Biotechnol. 2 (May), 9. 10.1186/1472-6750-2-9 12003642 PMC113741

[B10] Coquel A. S. Jacob J. P. Primet M. Demarez A. Dimiccoli M. Julou T. (2013). Localization of protein aggregation in Escherichia coli is governed by diffusion and nucleoid macromolecular crowding effect. PLoS Comput. Biol. 9 (4), e1003038. 10.1371/JOURNAL.PCBI.1003038 23633942 PMC3636022

[B11] Corso G. Mäger I. Lee Y. I. Görgens A. Bultema J. Giebel B. (2017). Reproducible and scalable purification of extracellular vesicles using combined bind-elute and size exclusion chromatography. Sci. Rep. 7 (1), 11561. 10.1038/S41598-017-10646-X 28912498 PMC5599601

[B12] Dennis J. J. Lafontaine E. R. Sokol P. A. (1996). Identification and characterization of the TolQRA genes of Pseudomonas aeruginosa. J. Bacteriol. 178 (24), 7059–7068. 10.1128/JB.178.24.7059-7068.1996 8955385 PMC178616

[B13] Dorward D. W. Garon C. F. (1990). DNA is packaged within membrane-derived vesicles of gram-negative but not gram-positive bacteria. Appl. Environ. Microbiol. 56 (6), 1960–1962. 10.1128/AEM.56.6.1960-1962.1990 16348232 PMC184538

[B14] Elkodous M. A. Sahoo S. Kumar R. Kumar R. (2023). Recent advances in modification of novel carbon-based composites: synthesis, properties, and biotechnological/biomedical applications. Chemico-Biological Interact. 379 (July), 110517. 10.1016/j.cbi.2023.110517 37149208

[B15] Ferrari G. Garaguso I. Adu-Bobie J. Doro F. Taddei A. R. Biolchi A. (2006). Outer membrane vesicles from group B **Neisseria meningitidis** Δ**gna33** mutant: proteomic and immunological comparison with detergent‐derived outer membrane vesicles. Proteomics 6 (6), 1856–1866. 10.1002/PMIC.200500164 16456881

[B16] Fulcrand G. Dages S. Zhi X. Chapagain P. Gerstman B. S. Dunlap D. (2016). DNA supercoiling, a critical signal regulating the basal expression of the lac operon in Escherichia coli. Sci. Rep. 6 (January), 19243. 10.1038/SREP19243 26763930 PMC4725879

[B17] Gerding M. A. Ogata Y. Pecora N. D. Niki H. De Boer P. A. J. (2007). The trans-envelope tol-pal complex is part of the cell division machinery and required for proper outer-membrane invagination during cell constriction in E. Coli. Mol. Microbiol. 63 (4), 1008–1025. 10.1111/J.1365-2958.2006.05571.X 17233825 PMC4428343

[B18] Gerritzen M. J. H. Martens D. E. Wijffels R. H. van der Pol L. Stork M. (2017). Bioengineering bacterial outer membrane vesicles as vaccine platform. Biotechnol. Adv. 35 (5), 565–574. 10.1016/J.BIOTECHADV.2017.05.003 28522212

[B19] Guzmán-Sastoque P. Sotelo S. Esmeral N. P. Luz Albarracín S. Sutachan J.-J. Reyes L. H. (2024). Assessment of CRISPRa-mediated gdnf overexpression in an *in vitro* Parkinson’s disease model. Front. Bioeng. Biotechnol. 12 (August), 1420183. 10.3389/fbioe.2024.1420183 39175618 PMC11338903

[B20] Hoy B. Löwer M. Weydig C. Carra G. Tegtmeyer N. Geppert T. (2010). Helicobacter pylori HtrA is a new secreted virulence factor that cleaves E-cadherin to disrupt intercellular adhesion. EMBO Rep. 11 (10), 798–804. 10.1038/EMBOR.2010.114 20814423 PMC2948180

[B21] Jefferies D. Shearer J. Khalid S. (2019). Role of O-antigen in response to mechanical stress of the E. Coli outer membrane: insights from coarse-grained MD simulations. J. Phys. Chem. B 123 (17), 3567–3575. 10.1021/ACS.JPCB.8B12168 30971088

[B22] Khider M. Willassen N. P. Hansen H. (2018). The alternative sigma factor RpoQ regulates colony morphology, biofilm formation and motility in the fish pathogen aliivibrio salmonicida. BMC Microbiol. 18 (1), 116. 10.1186/s12866-018-1258-9 30208852 PMC6134601

[B23] Kim Oh Y. Park H. T. Dinh N. T. H. Jin Choi S. Lee J. Kim Ji H. (2017). Bacterial outer membrane vesicles suppress tumor by interferon-γ-mediated antitumor response. Nat. Commun. 8 (1), 626. 10.1038/s41467-017-00729-8 28931823 PMC5606984

[B24] Koebnik R. Locher K. P. Van Gelder P. (2000). Structure and function of bacterial outer membrane proteins: barrels in a nutshell. Mol. Microbiol. 37 (2), 239–253. 10.1046/J.1365-2958.2000.01983.X 10931321

[B25] Kulp A. Kuehn M. J. (2010). Biological functions and biogenesis of secreted bacterial outer membrane vesicles. Annu. Rev. Microbiol. 64 (October), 163–184. 10.1146/ANNUREV.MICRO.091208.073413 20825345 PMC3525469

[B26] Lee E. Y. Choi D. S. Kim K. P. Gho Y. S. (2008). Proteomics in gram-negative bacterial outer membrane vesicles. Mass Spectrom. Rev. 27 (6), 535–555. 10.1002/MAS.20175 18421767

[B27] Li M. Zhou H. Yang C. Wu Y. I. Zhou X. Liu H. (2020). Bacterial outer membrane vesicles as a platform for biomedical applications: an update. J. Control. Release Official J. Control. Release Soc. 323 (July), 253–268. 10.1016/J.JCONREL.2020.04.031 32333919

[B28] Li N. Wu M. Wang L. U. Tang M. Xin H. Deng K. (2024). Efficient isolation of outer membrane vesicles (OMVs) secreted by gram-negative bacteria via a novel gradient filtration method. Membranes 14 (6), 135. 10.3390/membranes14060135 38921502 PMC11205348

[B29] Li P. Peng T. Xiang T. Luo W. Liao W. Wei D.-D. (2023). Klebsiella pneumoniae outer membrane vesicles induce strong IL-8 expression via NF-κb activation in normal pulmonary bronchial cells. Int. Immunopharmacol. 121 (August), 110352. 10.1016/j.intimp.2023.110352 37354781

[B30] Li R. Liu Q. (2020). Engineered bacterial outer membrane vesicles as multifunctional delivery platforms. Front. Mater. 7 (July). 10.3389/fmats.2020.00202

[B31] Lindner A. B. Madden R. Demarez A. Stewart E. J. Taddei F. (2008). Asymmetric segregation of protein aggregates is associated with cellular aging and rejuvenation. Proc. Natl. Acad. Sci. U. S. A. 105 (8), 3076–3081. 10.1073/pnas.0708931105 18287048 PMC2268587

[B32] McBroom A. J. Johnson A. P. Vemulapalli S. Kuehn M. J. (2006). Outer membrane vesicle production by Escherichia coli is independent of membrane instability. J. Bacteriol. 188 (15), 5385–5392. 10.1128/JB.00498-06 16855227 PMC1540050

[B33] Nordin J. Z. Lee Yi Mäger I. Johansson H. J. Heusermann W. Wiklander O. P. B. (2015). Ultrafiltration with size-exclusion liquid chromatography for high yield isolation of extracellular vesicles preserving intact biophysical and functional properties. Nanomedicine Nanotechnol. Biol. Med. 11 (4), 879–883. 10.1016/J.NANO.2015.01.003 25659648

[B34] Ortseifen V. Viefhues M. Wobbe L. Grünberger A. (2020). Microfluidics for biotechnology: bridging gaps to foster microfluidic applications. Front. Bioeng. Biotechnol. 8 (November), 589074. 10.3389/fbioe.2020.589074 33282849 PMC7691494

[B35] Páez-Vélez C. Rivas R. E. Dussán J. (2019). Enhanced gold biosorption of Lysinibacillus sphaericus CBAM5 by encapsulation of bacteria in an alginate matrix. Metals 9 (8), 818. 10.3390/MET9080818

[B36] Palma V. Gutiérrez M. S. Vargas O. Parthasarathy R. Navarrete P. (2022). Methods to evaluate bacterial motility and its role in bacterial–host interactions. Microorganisms 10 (3), 563. 10.3390/microorganisms10030563 35336138 PMC8953368

[B37] Partridge J. D. Harshey R. M. (2020). Investigating flagella-driven motility in Escherichia coli by applying three established techniques in a Series. J. Vis. Exp. (159). 10.3791/61364 PMC8453667 32449734

[B38] Petiti M. Serrano B. Faure L. Lloubes R. Mignot T. Duché D. (2019). Tol energy-driven localization of pal and anchoring to the peptidoglycan promote outer-membrane constriction. J. Mol. Biol. 431 (17), 3275–3288. 10.1016/J.JMB.2019.05.039 31153904

[B39] Qing G. Gong N. Chen X. Chen J. Zhang H. Wang Y. (2019). Natural and engineered bacterial outer membrane vesicles. Biophys. Rep. 5 (4), 184–198. 10.1007/s41048-019-00095-6

[B40] Reimer S. L. Beniac D. R. Hiebert S. L. Booth T. F. Chong P. M. Westmacott G. R. (2021). Comparative analysis of outer membrane vesicle isolation methods with an Escherichia coli TolA mutant reveals a hypervesiculating phenotype with outer-inner membrane vesicle content. Front. Microbiol. 12 (March), 628801. 10.3389/fmicb.2021.628801 33746922 PMC7973035

[B41] Rinas U. Garcia-Fruitós E. Luis Corchero J. Vázquez E. Seras-Franzoso J. Villaverde A. (2017). Bacterial inclusion bodies: discovering their better half. Trends Biochem. Sci. 42 (9), 726–737. 10.1016/J.TIBS.2017.01.005 28254353

[B42] Rodríguez C. F. Andrade-Pérez V. Vargas M. C. Mantilla-Orozco A. Osma J. F. Reyes L. H. (2023). Breaking the clean room barrier: exploring low-cost alternatives for microfluidic devices. Front. Bioeng. Biotechnol. 11 (April), 1176557. 10.3389/fbioe.2023.1176557 37180035 PMC10172592

[B43] Rodríguez C. F. Báez-Suárez M. Muñoz-Camargo C. Reyes L. H. Osma J. F. Cruz J. C. 2024a). Zweifach–fung microfluidic device for efficient microparticle separation: cost-effective fabrication using CO2 laser-ablated PMMA. Micromachines 15 (7), 932. 10.3390/mi15070932 39064443 PMC11278838

[B44] Rodríguez C. F. Guzmán-Sastoque P. Gantiva-Diaz M. Gómez S. C. Quezada V. Muñoz-Camargo C. (2023). Low-cost inertial microfluidic device for microparticle separation: a laser-ablated pmma lab-on-a-chip approach without a cleanroom. HardwareX 16 (December), e00493. 10.1016/j.ohx.2023.e00493 38045919 PMC10689937

[B45] Rodríguez C. F. Guzmán-Sastoque P. Muñoz-Camargo C. Reyes L. H. Osma J. F. Cruz J. C. (2024b). Enhancing magnetic micro- and nanoparticle separation with a cost-effective microfluidic device fabricated by laser ablation of PMMA. Micromachines 15, 1057. 10.3390/mi15081057 39203709 PMC11356012

[B46] Rodriguez C. F. Laura Ortiz C. Kevin A. Giraldo R. Carolina Munoz C. Cruz J. C. (2021). “ *In silico* study of spheroids fusion through magnetic field gradients,” in 2021 IEEE 2nd international congress of biomedical engineering and bioengineering (CI-IB&BI) (IEEE), 1–9. 10.1109/CI-IBBI54220.2021.9626089

[B47] Roier S. Zingl F. G. Cakar F. Durakovic S. Kohl P. Eichmann T. O. (2016). A novel mechanism for the biogenesis of outer membrane vesicles in gram-negative bacteria. Nat. Commun. 7 (January), 10515. 10.1038/NCOMMS10515 26806181 PMC4737802

[B48] Rottem S. Leive L. (1977). Effect of variations in lipopolysaccharide on the fluidity of the outer membrane of Escherichia coli. J. Biol. Chem. 252 (6), 2077–2081. 10.1016/S0021-9258(18)71867-X 191452

[B49] Santini C. L. Bernadac A. Zhang M. Chanal A. Ize B. Blanco C. (2001). Translocation of jellyfish green fluorescent protein via the Tat system of Escherichia coli and change of its periplasmic localization in response to osmotic up-shock. J. Biol. Chem. 276 (11), 8159–8164. 10.1074/JBC.C000833200 11099493

[B50] Schwechheimer C. Kuehn M. J. (2015). Outer-membrane vesicles from gram-negative bacteria: biogenesis and functions. Nat. Rev. Microbiol. 13 (10), 605–619. 10.1038/nrmicro3525 26373371 PMC5308417

[B51] Scorza B. Colucci A. M. Maggiore L. Sanzone S. Rossi O. Ferlenghi I. (2012). High yield production process for Shigella outer membrane particles. PloS One 7 (6), e35616. 10.1371/JOURNAL.PONE.0035616 22701551 PMC3368891

[B52] Scorza F. B. Doro F. Rodríguez-Ortega M. J. Stella M. Liberatori S. Taddei A. R. (2008). Proteomics characterization of outer membrane vesicles from the extraintestinal pathogenic Escherichia coli DeltatolR IHE3034 mutant. Mol. and Cell. Proteomics MCP 7 (3), 473–485. 10.1074/MCP.M700295-MCP200 17982123

[B53] Shahcheraghi N. Golchin H. Sadri Z. Tabari Y. Borhanifar F. Makani S. (2022). Nano-biotechnology, an applicable approach for sustainable future. 3 Biotech. 12 (3), 65. 10.1007/s13205-021-03108-9 PMC8828840 35186662

[B54] Shaner N. C. Steinbach P. A. Tsien R. Y. (2005). A guide to choosing fluorescent proteins. Nat. Methods 2 (12), 905–909. 10.1038/NMETH819 16299475

[B55] Sharif E. Eftekhari Z. Mohit E. (2021). The effect of growth stage and isolation method on properties of ClearColi^TM^ outer membrane vesicles (OMVs). Curr. Microbiol. 78 (4), 1602–1614. 10.1007/s00284-021-02414-y 33687512

[B56] Soni A. Bhandari M. P. Tripathi G. K. Bundela P. Kumar Khiriya P. Khare P. S. (2023). Nano‐biotechnology in tumour and cancerous disease: a perspective review. J. Cell. Mol. Med. 27 (6), 737–762. 10.1111/jcmm.17677 36840363 PMC10002932

[B57] Szczepaniak J. Press C. Kleanthous C. (2020). The multifarious roles of tol-pal in gram-negative bacteria. FEMS Microbiol. Rev. 44 (4), 490–506. 10.1093/FEMSRE/FUAA018 32472934 PMC7391070

[B58] Tamar E. Koler M. Vaknin A. (2016). The role of motility and chemotaxis in the bacterial colonization of protected surfaces. Sci. Rep. 6 (1), 19616. 10.1038/srep19616 26792493 PMC4726332

[B59] Tegel H. Ottosson J. Hober S. (2011). Enhancing the protein production levels in *Escherichia coli* with a strong promoter. FEBS J. 278 (5), 729–739. 10.1111/j.1742-4658.2010.07991.x 21205203

[B60] Thomas J. D. Daniel R. A. Errington J. Robinson C. (2001). Export of active green fluorescent protein to the periplasm by the twin-arginine translocase (Tat) pathway in Escherichia coli. Mol. Microbiol. 39 (1), 47–53. 10.1046/J.1365-2958.2001.02253.X 11123687

[B61] Tsien R. Y. (1998). The green fluorescent protein. Annu. Rev. Biochem. 67, 509–544. 10.1146/ANNUREV.BIOCHEM.67.1.509 9759496

[B62] Valderrama J. D. Gutierrez F. R. S. (2018). “Engineering of bacterial outer membrane vesicles: potential applications for the development of vaccines,” in Lipid nanocarriers for drug targeting. Elsevier Inc. 10.1016/B978-0-12-813687-4.00005-0

[B63] Walburger A. Lazdunski C. Corda Y. (2002). The tol/pal system function requires an interaction between the C-terminal domain of TolA and the N-terminal domain of TolB. Mol. Microbiol. 44 (3), 695–708. 10.1046/J.1365-2958.2002.02895.X 11994151

[B64] Wang J. Ma W. Wang X. (2021). Insights into the structure of Escherichia coli outer membrane as the target for engineering microbial cell factories. Microb. Cell Factories 20 (1), 73–17. 10.1186/S12934-021-01565-8 PMC7980664 33743682

[B65] Weigand R. A. Rothfield L. I. (1976). Genetic and physiological classification of periplasmic-leaky mutants of Salmonella typhimurium. J. Bacteriol. 125 (1), 340–345. 10.1128/JB.125.1.340-345.1976 812862 PMC233368

[B66] Winkler J. Seybert A. König L. Pruggnaller S. Haselmann U. Sourjik V. (2010). Quantitative and spatio-temporal features of protein aggregation in Escherichia coli and consequences on protein quality control and cellular ageing. EMBO J. 29 (5), 910–923. 10.1038/EMBOJ.2009.412 20094032 PMC2837176

[B67] Wu W. Xing L. Zhou B. Lin Z. (2011). Active protein aggregates induced by terminally attached self-assembling peptide ELK16 in Escherichia coli. Microb. Cell Factories 10 (February), 9. 10.1186/1475-2859-10-9 PMC3045283 21320350

[B68] Yakhnina A. A. Bernhardt T. G. (2020). The tol-pal system is required for peptidoglycan-cleaving enzymes to complete bacterial cell division. Proc. Natl. Acad. Sci. U. S. A. 117 (12), 6777–6783. 10.1073/pnas.1919267117 32152098 PMC7104345

[B69] Zhao L. Lu L. I. Wang A. Zhang H. Huang M. Wu H. (2020). Nano-biotechnology in agriculture: use of nanomaterials to promote plant growth and stress tolerance. J. Agric. Food Chem. 68 (7), 1935–1947. 10.1021/acs.jafc.9b06615 32003987

