# Supplementary material for: Production and purification of outer membrane vesicles encapsulating green fluorescent protein from Escherichia coli: a step towards scalable OMV technologies
Source: Front Bioeng Biotechnol. 2024 Nov 14;12:1436352. doi: 10.3389/fbioe.2024.1436352 (PMC11602331; doi:10.3389/fbioe.2024.1436352)
Supplement: Supplementary file 1 [file DataSheet1.docx]

Supplementary Material

Efficient Production and Purification of Outer Membrane Vesicles (OMVs) Encapsulating Green Fluorescent Protein from *Escherichia coli*: A Step towards Scalable OMV Technologies

Julian Daniel Torres-Vanegas (J.D.T-V) ^1^, Nicolas Rincon-Tellez (N.R-T) ^1,2^, Paula Guzmán-Sastoque (P. G-S)^4^, Juan D. Valderrama-Rincon (J.D.V-R)^3^, Juan C. Cruz (J.C.C) ^1,4*^, Luis H. Reyes (L.H.R) ^1*^

^1^Grupo de Diseño de Productos y Procesos (GDPP), Department of Chemical and Food Engineering, Universidad de Los Andes, Bogota D.C, Colombia

^2^Department of Biological Sciences, Universidad de Los Andes, Bogota D.C, Colombia

^3^Grupo GRESIA, Department of Environmental Engineering, Universidad Antonio Nariño, Bogota D.C, Colombia

^4^Department of Biomedical Engineering, Universidad de Los Andes, Bogota D.C, Colombia

*** Correspondence:**Juan C. Cruz; Luis H. Reyes
[jc.cruz@uniandes.edu.co](mailto:jc.cruz@uniandes.edu.co); [lh.reyes@uniandes.edu.co](mailto:lh.reyes@uniandes.edu.co)


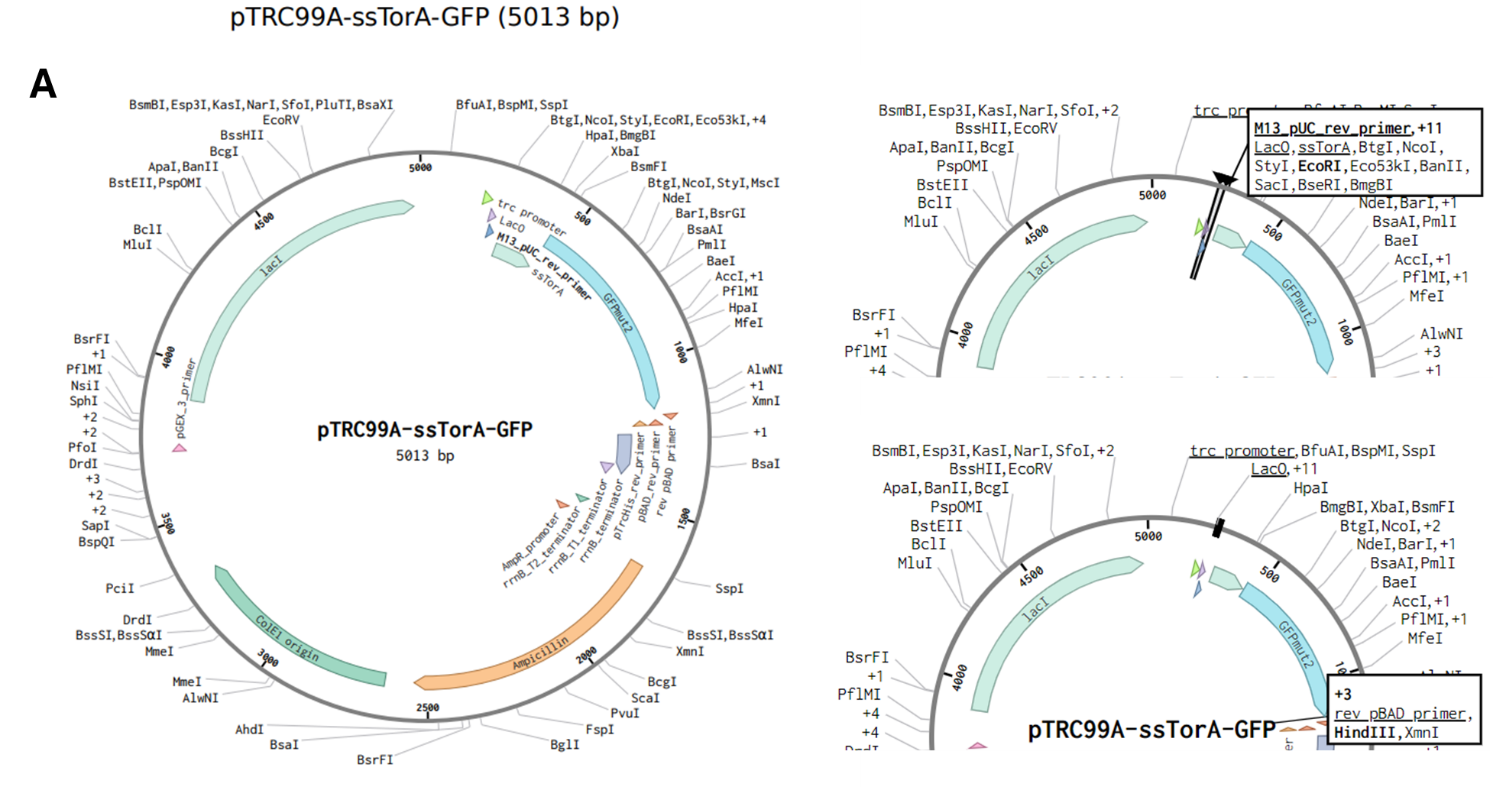


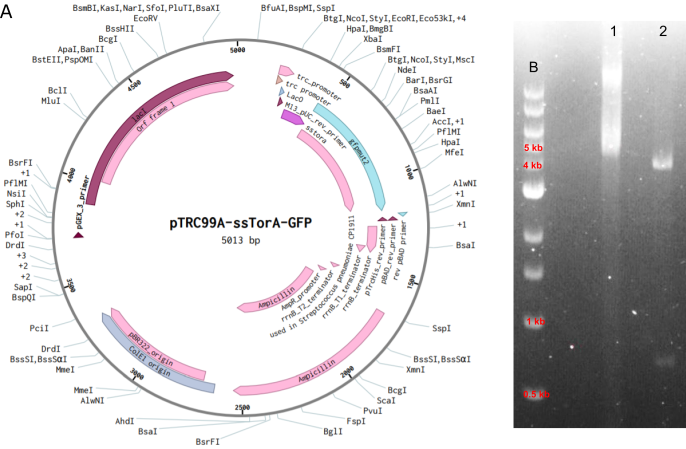


**Figure S1.** Corroboration of the presence of the plasmid pTRC99A-ssTorA-GFP via digestion with EcoRI and HindIII: A) Schematic of the plasmid map. B) Lane 1, Undigested DNA. Lane 2, Digested DNA


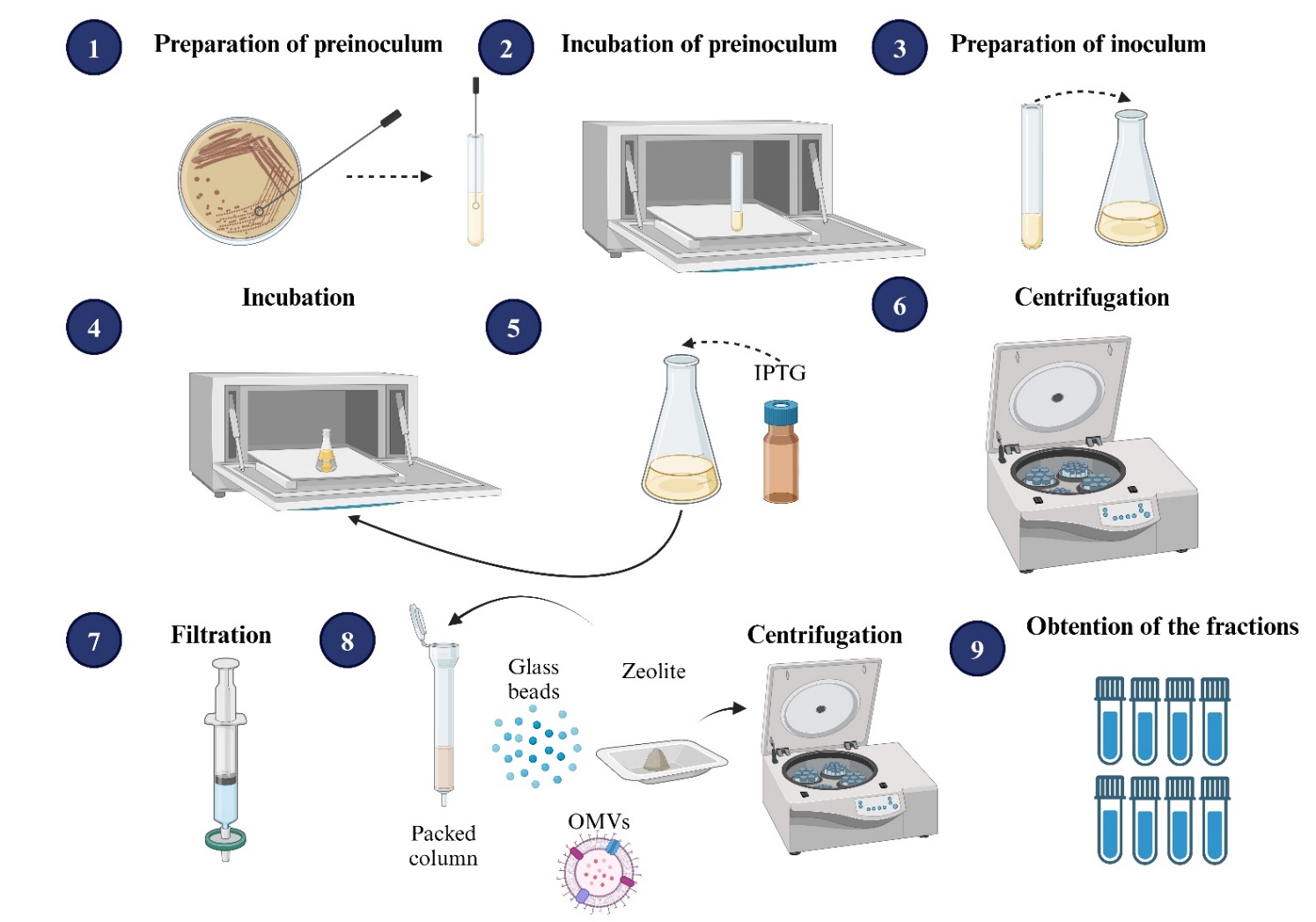


**Figure S2.** Schematic of the experimental procedure for the synthesis and purification of OMVs.


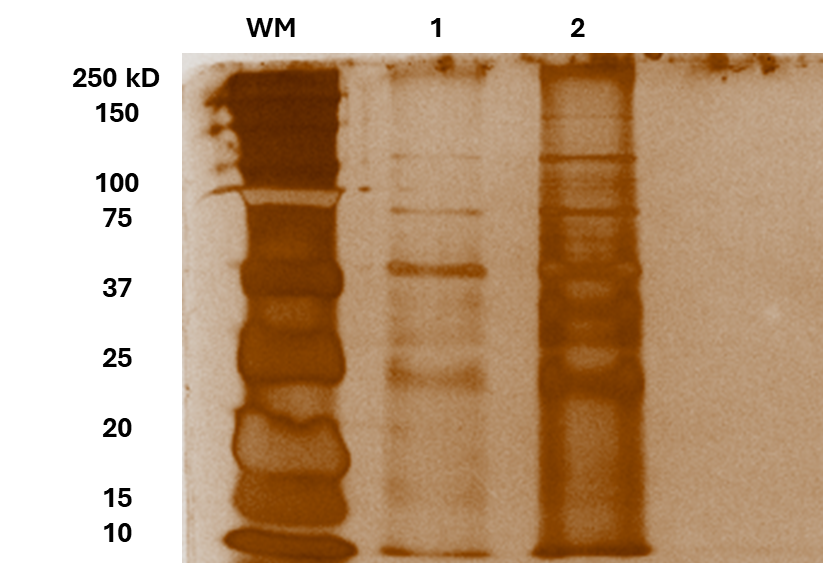


**Figure S3.** SDS-PAGE conducted with silver staining. Lane 1: Fraction #4 of SEC after lysis with 1% *v/v* Triton X-100. Lane 2: OMVs broken with 1% *v/v* Triton X-100 before SEC purification.

**Plasmid sequence**

LOCUS pTRC99A_sstora_gfp 5013 bp ds-DNA circular

DEFINITION pTrc99A Sequence sequence

KEYWORDS .

SOURCE pTrc99A Sequence sequence

ORGANISM other sequences; artificial sequences; vectors.

COMMENT See Addgene Vector Database pTrc99A Sequence sequence at

www.addgene.org

COMMENT ApEinfo:methylated:1

FEATURES Location/Qualifiers

promoter 193..266

/ApEinfo_graphicformat=arrow_data {{0 1 2 0 0 -1} {} 0}

width 5 offset 0

/label=trc_promoter

/ApEinfo_fwdcolor=pink

/ApEinfo_revcolor=pink

misc_feature 235..257

/ApEinfo_graphicformat=arrow_data {{0 1 2 0 0 -1} {} 0}

width 5 offset 0

/label=M13_pUC_rev_primer

/ApEinfo_fwdcolor=#800040

/ApEinfo_revcolor=#800000

misc_feature complement(1196..1213)

/ApEinfo_graphicformat=arrow_data {{0 1 2 0 0 -1} {} 0}

width 5 offset 0

/label=pBAD_rev_primer

/ApEinfo_fwdcolor=#800040

/ApEinfo_revcolor=#800000

misc_feature complement(1196..1213)

/ApEinfo_graphicformat=arrow_data {{0 1 2 0 0 -1} {} 0}

width 5 offset 0

/label=pTrcHis_rev_primer

/ApEinfo_fwdcolor=#800040

/ApEinfo_revcolor=#800000

terminator 1246..1403

/ApEinfo_graphicformat=arrow_data {{0 1 2 0 0 -1} {} 0}

width 5 offset 0

/label=rrnB_terminator

/ApEinfo_fwdcolor=pink

/ApEinfo_revcolor=pink

terminator 1369..1412

/ApEinfo_graphicformat=arrow_data {{0 1 2 0 0 -1} {} 0}

width 5 offset 0

/label=rrnB_T1_terminator

/ApEinfo_fwdcolor=pink

/ApEinfo_revcolor=pink

terminator 1544..1571

/ApEinfo_graphicformat=arrow_data {{0 1 2 0 0 -1} {} 0}

width 5 offset 0

/label=rrnB_T2_terminator

/ApEinfo_fwdcolor=pink

/ApEinfo_revcolor=pink

promoter 1613..1641

/ApEinfo_graphicformat=arrow_data {{0 1 2 0 0 -1} {} 0}

width 5 offset 0

/label=AmpR_promoter

/ApEinfo_fwdcolor=pink

/ApEinfo_revcolor=pink

CDS 1683..2543

/gene="Ampicillin"

/note="Orf frame 3"

/translation="MSIQHFRVALIPFFAAFCLPVFAHPETLVKVKDAEDQLGARVGY

IELDLNSGKILESFRPEERFPMMSTFKVLLCGAVLSRVDAGQEQLGRRIHYSQNDLVE

YSPVTEKHLTDGMTVRELCSAAITMSDNTAANLLLTTIGGPKELTAFLHNMGDHVTRL

DRWEPELNEAIPNDERDTTMPTAMATTLRKLLTGELLTLASRQQLIDWMEADKVAGPL

LRSALPAGWFIADKSGAGERGSRGIIAALGPDGKPSRIVVIYTTGSQATMDERNRQIA

EIGASLIKHW*"

/ApEinfo_graphicformat=arrow_data {{0 1 2 0 0 -1} {} 0}

width 5 offset 0

/label=Ampicillin

/ApEinfo_fwdcolor=pink

/ApEinfo_revcolor=pink

gene 1683..2543

/gene="Ampicillin"

/ApEinfo_graphicformat=arrow_data {{0 1 2 0 0 -1} {} 0}

width 5 offset 0

/label=Ampicillin(1)

/ApEinfo_label=Ampicillin

/ApEinfo_fwdcolor=pink

/ApEinfo_revcolor=pink

rep_origin 2698..3317

/ApEinfo_graphicformat=arrow_data {{0 1 2 0 0 -1} {} 0}

width 5 offset 0

/label=pBR322_origin

/ApEinfo_fwdcolor=pink

/ApEinfo_revcolor=pink

misc_feature 3714..3736

/ApEinfo_graphicformat=arrow_data {{0 1 2 0 0 -1} {} 0}

width 5 offset 0

/label=pGEX_3_primer

/ApEinfo_fwdcolor=#800040

/ApEinfo_revcolor=#800000

misc_feature 3883..4974

/ApEinfo_graphicformat=arrow_data {{0 1 2 0 0 -1} {} 0}

width 5 offset 0

/label=lacI

/ApEinfo_fwdcolor=#800040

/ApEinfo_revcolor=#800000

CDS 4015..4974

/translation="MAELNYIPNRVAQQLAGKQSLLIGVATSSLALHAPSQIVAAIKS

RADQLGASVVVSMVERSGVEACKAAVHNLLAQRVSGLIINYPLDDQDAIAVEAACTNV

PALFLDVSDQTPINSIIFSHEDGTRLGVEHLVALGHQQIALLAGPLSSVSARLRLAGW

HKYLTRNQIQPIAEREGDWSAMSGFQQTMQMLNEGIVPTAMLVANDQMALGAMRAITE

SGLRVGADISVVGYDDTEDSSCYIPPSTTIKQDFRLLGQTSVDRLLQLSQGQAVKGNQ

LLPVSLVKRKTTLAPNTQTASPRALADSLMQLARQVSRLESGQ*"

/ApEinfo_graphicformat=arrow_data {{0 1 2 0 0 -1} {} 0}

width 5 offset 0

/label=Orf frame 1

/ApEinfo_fwdcolor=pink

/ApEinfo_revcolor=pink

source 444..1136

/organism="synthetic construct"

/mol_type="other DNA"

/db_xref="taxon:32630"

/PCR_primers="fwd_name: SA81, fwd_seq:

atattctctttgagtcctgctctgg, rev_name: SA86, rev_seq:

caacgatatctttgactaactgt"

/note="used in Streptococcus pneumoniae CP1911"

/ApEinfo_graphicformat=arrow_data {{0 1 2 0 0 -1} {} 0}

width 5 offset 0

/label=used in Streptococcus pneumoniae CP1911

/ApEinfo_fwdcolor=pink

/ApEinfo_revcolor=pink

misc_feature 1180..1197

/ApEinfo_graphicformat=arrow_data {{0 1 2 0 0 -1} {} 0}

width 5 offset 0

/label=rev pBAD primer

/ApEinfo_fwdcolor=cyan

/ApEinfo_revcolor=cyan

misc_feature 444..1157

/ApEinfo_graphicformat=arrow_data {{0 1 2 0 0 -1} {} 0}

width 5 offset 0

/label=gfpmut2

/ApEinfo_fwdcolor=cyan

/ApEinfo_revcolor=green

misc_feature 282..437

/ApEinfo_graphicformat=arrow_data {{0 1 2 0 0 -1} {} 0}

width 5 offset 0

/label=sstora

/ApEinfo_fwdcolor=#c92ed2

/ApEinfo_revcolor=#c92ed2

ORIGIN

1 gtttgacagc ttatcatcga ctgcacggtg caccaatgct tctggcgtca ggcagccatc

61 ggaagctgtg gtatggctgt gcaggtcgta aatcactgca taattcgtgt cgctcaaggc

121 gcactcccgt tctggataat gttttttgcg ccgacatcat aacggttctg gcaaatattc

181 tgaaatgagc tgttgacaat taatcatccg gctcgtataa tgtgtggaat tgtgagcgga

241 taacaatttc acacaggaaa cagaccatgg aattcgagct cttaaagagg agaaaggtca

301 tgAACAATAA CGATCTCTTT CAGGCATCAC GTCGGCGTTT TCTGGCACAA CTCGGCGGCT

361 TAACCGTCGC CGGGATGCTG GGGCCGTCAT TGTTAACGCC GCGACGTGCG ACTGCGGCGC

421 AAGCGGCGAC TGACGCTTCT AGAagtaaag gagaagaact tttcactgga gttgtcccaa

481 ttcttgttga attagatggc gatgttaatg ggcaaaaatt ctctgtcagt ggagagggtg

541 aaggtgatgc aacatacgga aaacttaccc ttaaatttat ttgcactact gggaagctac

601 ctgttccatg gccaacactt gtcactactt tcgcgtatgg tcttcaatgc tttgcgagat

661 acccagatca tatgaaacag catgactttt tcaagagtgc catgcccgaa ggttatgtac

721 aggaaagaac tatattttac aaagatgacg ggaactacaa gacacgtgct gaagtcaagt

781 ttgaaggtga tacccttgtt aatagaatcg agttaaaagg tattgatttt aaagaagatg

841 gaaacattct tggacacaaa atggaataca actataactc acataatgta tacatcatgg

901 cagacaaacc aaagaatgga atcaaagtta acttcaaaat tagacacaac attaaagatg

961 gaagcgttca attagcagac cattatcaac aaaatactcc aattggcgat ggccctgtcc

1021 ttttaccaga caaccattac ctgtccacac aatctgccct ttccaaagat cccaacgaaa

1081 agagagatca catgatcctt cttgagtttg taacagctgc tgggattaca catggcatgg

1141 atgaactata caaatagaag cttggctgtt ttggcggatg agagaagatt ttcagcctga

1201 tacagattaa atcagaacgc agaagcggtc tgataaaaca gaatttgcct ggcggcagta

1261 gcgcggtggt cccacctgac cccatgccga actcagaagt gaaacgccgt agcgccgatg

1321 gtagtgtggg gtctccccat gcgagagtag ggaactgcca ggcatcaaat aaaacgaaag

1381 gctcagtcga aagactgggc ctttcgtttt atctgttgtt tgtcggtgaa cgctctcctg

1441 agtaggacaa atccgccggg agcggatttg aacgttgcga agcaacggcc cggagggtgg

1501 cgggcaggac gcccgccata aactgccagg catcaaatta agcagaaggc catcctgacg

1561 gatggccttt ttgcgtttct acaaactctt tttgtttatt tttctaaata cattcaaata

1621 tgtatccgct catgagacaa taaccctgat aaatgcttca ataatattga aaaaggaaga

1681 gtatgagtat tcaacatttc cgtgtcgccc ttattccctt ttttgcggca ttttgccttc

1741 ctgtttttgc tcacccagaa acgctggtga aagtaaaaga tgctgaagat cagttgggtg

1801 cacgagtggg ttacatcgaa ctggatctca acagcggtaa gatccttgag agttttcgcc

1861 ccgaagaacg ttttccaatg atgagcactt ttaaagttct gctatgtggc gcggtattat

1921 cccgtgttga cgccgggcaa gagcaactcg gtcgccgcat acactattct cagaatgact

1981 tggttgagta ctcaccagtc acagaaaagc atcttacgga tggcatgaca gtaagagaat

2041 tatgcagtgc tgccataacc atgagtgata acactgcggc caacttactt ctgacaacga

2101 tcggaggacc gaaggagcta accgcttttt tgcacaacat gggggatcat gtaactcgcc

2161 ttgatcgttg ggaaccggag ctgaatgaag ccataccaaa cgacgagcgt gacaccacga

2221 tgcctacagc aatggcaaca acgttgcgca aactattaac tggcgaacta cttactctag

2281 cttcccggca acaattaata gactggatgg aggcggataa agttgcagga ccacttctgc

2341 gctcggccct tccggctggc tggtttattg ctgataaatc tggagccggt gagcgtgggt

2401 ctcgcggtat cattgcagca ctggggccag atggtaagcc ctcccgtatc gtagttatct

2461 acacgacggg gagtcaggca actatggatg aacgaaatag acagatcgct gagataggtg

2521 cctcactgat taagcattgg taactgtcag accaagttta ctcatatata ctttagattg

2581 atttaaaact tcatttttaa tttaaaagga tctaggtgaa gatccttttt gataatctca

2641 tgaccaaaat cccttaacgt gagttttcgt tccactgagc gtcagacccc gtagaaaaga

2701 tcaaaggatc ttcttgagat cctttttttc tgcgcgtaat ctgctgcttg caaacaaaaa

2761 aaccaccgct accagcggtg gtttgtttgc cggatcaaga gctaccaact ctttttccga

2821 aggtaactgg cttcagcaga gcgcagatac caaatactgt ccttctagtg tagccgtagt

2881 taggccacca cttcaagaac tctgtagcac cgcctacata cctcgctctg ctaatcctgt

2941 taccagtggc tgctgccagt ggcgataagt cgtgtcttac cgggttggac tcaagacgat

3001 agttaccgga taaggcgcag cggtcgggct gaacgggggg ttcgtgcaca cagcccagct

3061 tggagcgaac gacctacacc gaactgagat acctacagcg tgagctatga gaaagcgcca

3121 cgcttcccga agggagaaag gcggacaggt atccggtaag cggcagggtc ggaacaggag

3181 agcgcacgag ggagcttcca gggggaaacg cctggtatct ttatagtcct gtcgggtttc

3241 gccacctctg acttgagcgt cgatttttgt gatgctcgtc aggggggcgg agcctatgga

3301 aaaacgccag caacgcggcc tttttacggt tcctggcctt ttgctggcct tttgctcaca

3361 tgttctttcc tgcgttatcc cctgattctg tggataaccg tattaccgcc tttgagtgag

3421 ctgataccgc tcgccgcagc cgaacgaccg agcgcagcga gtcagtgagc gaggaagcgg

3481 aagagcgcct gatgcggtat tttctcctta cgcatctgtg cggtatttca caccgcatat

3541 ggtgcactct cagtacaatc tgctctgatg ccgcatagtt aagccagtat acactccgct

3601 atcgctacgt gactgggtca tggctgcgcc ccgacacccg ccaacacccg ctgacgcgcc

3661 ctgacgggct tgtctgctcc cggcatccgc ttacagacaa gctgtgaccg tctccgggag

3721 ctgcatgtgt cagaggtttt caccgtcatc accgaaacgc gcgaggcagc agatcaattc

3781 gcgcgcgaag gcgaagcggc atgcatttac gttgacacca tcgaatggtg caaaaccttt

3841 cgcggtatgg catgatagcg cccggaagag agtcaattca gggtggtgaa tgtgaaacca

3901 gtaacgttat acgatgtcgc agagtatgcc ggtgtctctt atcagaccgt ttcccgcgtg

3961 gtgaaccagg ccagccacgt ttctgcgaaa acgcgggaaa aagtggaagc ggcgatggcg

4021 gagctgaatt acattcccaa ccgcgtggca caacaactgg cgggcaaaca gtcgttgctg

4081 attggcgttg ccacctccag tctggccctg cacgcgccgt cgcaaattgt cgcggcgatt

4141 aaatctcgcg ccgatcaact gggtgccagc gtggtggtgt cgatggtaga acgaagcggc

4201 gtcgaagcct gtaaagcggc ggtgcacaat cttctcgcgc aacgcgtcag tgggctgatc

4261 attaactatc cgctggatga ccaggatgcc attgctgtgg aagctgcctg cactaatgtt

4321 ccggcgttat ttcttgatgt ctctgaccag acacccatca acagtattat tttctcccat

4381 gaagacggta cgcgactggg cgtggagcat ctggtcgcat tgggtcacca gcaaatcgcg

4441 ctgttagcgg gcccattaag ttctgtctcg gcgcgtctgc gtctggctgg ctggcataaa

4501 tatctcactc gcaatcaaat tcagccgata gcggaacggg aaggcgactg gagtgccatg

4561 tccggttttc aacaaaccat gcaaatgctg aatgagggca tcgttcccac tgcgatgctg

4621 gttgccaacg atcagatggc gctgggcgca atgcgcgcca ttaccgagtc cgggctgcgc

4681 gttggtgcgg atatctcggt agtgggatac gacgataccg aagacagctc atgttatatc

4741 ccgccgtcaa ccaccatcaa acaggatttt cgcctgctgg ggcaaaccag cgtggaccgc

4801 ttgctgcaac tctctcaggg ccaggcggtg aagggcaatc agctgttgcc cgtctcactg

4861 gtgaaaagaa aaaccaccct ggcgcccaat acgcaaaccg cctctccccg cgcgttggcc

4921 gattcattaa tgcagctggc acgacaggtt tcccgactgg aaagcgggca gtgagcgcaa

4981 cgcaattaat gtgagttagc gcgaattgat ctg
